# Characterisation of Hypertensive Patients with Improved Endothelial Function after Dark Chocolate Consumption

**DOI:** 10.1155/2013/985087

**Published:** 2013-03-05

**Authors:** Jenifer d'El-Rei, Ana Rosa Cunha, Adriana Burlá, Marcelo Burlá, Wille Oigman, Mario Fritsch Neves, Agostino Virdis, Fernanda Medeiros

**Affiliations:** ^1^Faculty of Nutrition, Federal University of Rio de Janeiro, Rua Mariz e Barros 775, 20270-004 Rio de Janeiro, Rj, Brazil; ^2^Department of Clinical Medicine, State University of Rio de Janeiro, Avenida Vinte e Oito de Setembro, 77, Sala 329, 20551-030 Rio de Janeiro, Rj, Brazil; ^3^Department of Clinical and Experimental Medicine, University of Pisa, via Roma 67, 56126 Pisa, Italy

## Abstract

Recent findings indicate an inverse relationship between cardiovascular disease and consumption of flavonoids. We aimed to identify clinical and vascular parameters of treated hypertensive who present beneficial effects of dark chocolate for one-week period on vascular function. Twenty-one hypertensive subjects, aged 40–65 years, were included in a prospective study with measurement of blood pressure (BP), brachial flow-mediated dilatation (FMD), peripheral arterial tonometry, and central hemodynamic parameters. These tests were repeated after seven days of eating dark chocolate 75 g/day. Patients were divided according to the response in FMD: responders (*n* = 12) and nonresponders (*n* = 9). The responder group presented lower age (54 ± 7 versus 61 ± 6 years, *P* = 0.037), Framingham risk score (FRS) (2.5 ± 1.8 versus 8.1 ± 5.1%, *P* = 0.017), values of peripheral (55 ± 9 versus 63 ± 5 mmHg, *P* = 0.041), and central pulse pressure (PP) (44 ± 10 versus 54 ± 6 mmHg, *P* = 0.021). FMD response showed negative correlation with FRS (*r* = −0.60, *P* = 0.014), baseline FMD (*r* = −0.54, *P* = 0.011), baseline reactive hyperemia index (RHI; *r* = −0.56, *P* = 0.008), and central PP (*r* = −0.43, *P* = 0.05). However, after linear regression analysis, only FRS and baseline RHI were associated with FMD response. In conclusion, one-week dark chocolate intake significantly improved endothelial function and reduced BP in younger hypertensive with impaired endothelial function in spite of lower cardiovascular risk.

## 1. Introduction

Hypertension notably contributes to the worldwide cardiovascular morbidity and mortality. Hypertensive disease seems to have a complex association with endothelial dysfunction, a phenotypical alteration of the vascular endothelium that precedes the development of adverse cardiovascular events and anticipates future cardiovascular risk [1]. Several studies have confirmed the connection between hypertension and abnormal endothelial function in the peripheral, coronary, and renal circulation, suggesting an important mechanism, whereby hypertension promotes the development and progression of vascular disease [1–4]. Given that endothelial dysfunction may be reversible, early detection of this disorder may have therapeutic and prognostic implications [5, 6].

Lifestyle modifications, including dietary habits, have substantial effects on risk factors for cardiovascular disease such as hypertension [7]. Epidemiological evidence demonstrates that a diet rich in fruits and vegetables promotes heart and vascular health [8–10]. These beneficial effects have been largely ascribed to their content in flavonoids. These compounds are synthesized in many edible plants and remain present when plants are processed to foods. Grapes, wine, cocoa and chocolate, teas, and soy are among the most important sources of flavonoids in the human diet. A significant number of studies have been carried out in humans, analyzing the effect of foods rich in flavonoids on the presence and progression of risk factors associated with cardiovascular disease. Cocoa derived products have been thoroughly studied and demonstrated to be efficient improving endothelial function and decreasing blood pressure (BP) [11–14]. 

Some individuals may have positive health benefits when chocolate is ingested in moderation as part of a balanced diet [15]. Thus, the aim of this study was to identify clinical and vascular parameters of treated hypertensive patients who present beneficial effects of dark chocolate on vascular function for one-week period.

## 2. Materials and Methods

### 2.1. Study Population

Twenty-one hypertensive patients recruited from Pedro Ernesto Hospital on stable drug therapy over at least 4 weeks, aged 40–65 years, were included in a prospective study. Exclusion criteria were evidence of secondary hypertension, body mass index ≥ 35 Kg/m², coronary artery disease, kidney or thyroid disease, hormone replacement therapy, diabetes or impaired tolerance glucose, severe dyslipidemia (LDL-cholesterol ≥ 4.14 mmol/L and/or triglycerides ≥ 3.39 mmol/L), use of lipid-lowering drugs, using restricted diets (vegetarian, carbohydrate restriction), allergy to chocolate or cocoa, and use of nutritional supplements (vitamins, minerals) for up to seven days before the study beginning. The protocol was approved by the local Ethics Committee Research (2415-CEP/HUPE), and all patients gave written informed consent. This study was registered in Clinicaltrials.gov (NCT01314924).

### 2.2. Flow-Mediated Dilation (FMD) of Brachial Artery

After overnight fasting, without smoking or drinking coffee for a least 12 h before tests, patients were examined in the supine position in a dark, quiet, and air-conditioned room (22–24°C). A linear-array transducer operating at 10 MHz was used to acquire longitudinal images of the right brachial artery. A standard BP cuff was positioned around the right arm, 5 cm above the antecubital fossa. After obtaining baseline images of end-diastolic diameter of the brachial artery, the cuff was inflated to 50 mmHg above systolic BP for 5 minutes. Longitudinal images of the brachial artery were captured continuously for 30, 60, and 90 seconds after cuff deflation to document the vasodilator response. FMD was calculated as the percentage change in diameter from the baseline value to the peak value after cuff release. 

### 2.3. Study Design

Volunteer patients meeting the selection criteria underwent clinical and laboratory evaluation. For laboratory tests and vascular assessment, patients were asked to fast for eight to twelve hours. At the first visit, patients were instructed to maintain the habitual physical activity, usual diet, but avoiding consumption of foods rich in flavonoids (apples, grapes, wine, tea, and chocolate) during the intervention. All patients then received 75 g of dark chocolate with 70% cocoa, rich in flavonoids, daily for seven days.

After 1-week consumption of dark chocolate and reexamination, patients were divided according to the response in brachial FMD into responders (RESP) when there was at least an increase of 1% in FMD and nonresponders (N-RESP) when this improvement in FMD was not observed.

Total polyphenol content of the chocolate used in this study was measured by spectrophotometry in the Laboratory of Pharmacology at our institution. We found 42.7 mg of polyphenols/g of dark chocolate, resulting in 3202 mg of polyphenols in the total daily dose.

### 2.4. Clinical and Laboratory Analyses

Office brachial systolic and diastolic BP were measured in the sitting position using a validated electronic device (HEM-705CP, Omron Healthcare Inc., IL, USA) with an appropriate cuff size. Three consecutive readings, one minute apart, were obtained, and the average value was used as the clinic BP. 

Routine biochemical assessment included fasting glucose and lipid profile (total cholesterol, HDL-cholesterol, triglycerides, and LDL-cholesterol calculated by Friedewald formula). The Framingham risk score was estimated for each patient [16].

### 2.5. Peripheral Arterial Tonometry (PAT)

Endothelial function was also assessed by PAT using the Endo-PAT2000 device (Itamar Medical Ltd., Caesarea, Israel). PAT is a noninvasive technique, to assess peripheral microvascular endothelial function by measuring changes in digital arterial pulse volume during reactive hyperemia [5].

Probes were placed on index finger of each hand, and a BP cuff was placed on nondominant (study arm), while the contralateral arm served as a control. Continuous recording of pulsatile blood volume responses from both hands was initiated. After a 10 min equilibration period, the BP cuff on the study arm was inflated to 60 mmHg above systolic pressure. The changes in arterial tone were elicited by a standard 5 min occlusion of the brachial artery. The cuff was then deflated to induce reactive hyperemia, while PAT recording was continued. Reactive hyperemia index (RHI) was analysed by a computer in an operator-independent manner. 

### 2.6. Central Hemodynamic Parameters

Radial artery applanation tonometry and pulse wave analysis were carried out to derive central BP and other parameters using dedicated system SphygmoCor v.7 (SphygmoCor, AtCor Medical Inc., USA). This method generates central aortic pressure waveforms from the radial pressure waveform using a previously validated transfer function. The central pressure waves were analyzed to identify the outgoing and reflected components and to calculate the augmentation index (Aix), that is, the proportion of the central pulse pressure that is attributable to pulse wave reflection which is calculated as augmentation pressure (AP).

### 2.7. Sample Size Calculation

One goal of the proposed study is to test the null hypothesis that the mean difference (or change) within pairs is zero. The criterion for significance (alpha) has been set at 0,050. The test is 2 tailed, which means that an effect in either direction will be interpreted. With the proposed sample size of 20 pairs of cases, the study will have power of 80% to yield a statistically significant result.

### 2.8. Statistical Analysis

The results were expressed as mean ± standard deviation (SD) or as percentages when appropriate. In the intragroup analysis, continuous variables obtained before and after chocolate consumption were compared by paired *t*-test with a confidence interval of 95%, and the value of *P* < 0.05 was considered statistically significant. Student's *t*-test for independent samples was used for intergroup analysis. Pearson coefficient was obtained in correlation tests between continuous variables. Multiple linear regression, stepwise model, was done considering FMD response as dependent variable and those significantly correlated to this parameter as independent variables. Statistical analysis was performed using the Statistical Package for Social Sciences (SPSS, Inc., Chicago, IL) version 18.0.

## 3. Results

Twenty-four eligible patients were identified for this study. One patient was excluded due to high levels of fasting glucose, and another 2 patients failed to complete the intervention period. Thus, 21 individuals completed the study of whom 16 (76%) were female. Baseline characteristics and vascular profile of the study population are given in Table 1. A moderate consumption (twice a week) of alcohol was reported by 38% of patients. Regarding regular physical activity, only 4 patients (19%) were physically active on a regular basis.

Data on drug therapy show that classes of antihypertensive drugs used by patients were thiazide diuretics (95%), angiotensin receptor blocker (10%), angiotensin converting enzyme inhibitor (5%), and calcium channel antagonist (5%). From the total sample, 81% were on monotherapy (76% on thiazides), and 19% were on a combination of two drugs.

The clinical and lipid profiles of responders and nonresponders are shown in Table 1. Mean age and Framingham risk score were significantly lower among responders. Who also had greater impairment of endothelial function evidenced by decreased baseline brachial FMD and RHI. In addition, responder group presented significantly lower brachial and aortic pulse pressure than nonresponders (Table 2).

Besides improvement in FMD, the responder group presented significant changes in clinical parameters such as systolic (140 ± 13 versus 131 ± 10 mmHg, *P* < 0.05) and diastolic (85 ± 7 versus 82 ± 8 mmHg, *P* = 0.05) BP and in mean arterial pressure (103 ± 9 versus 98 ± 9 mmHg, *P* < 0.01).

**Figure 1:**
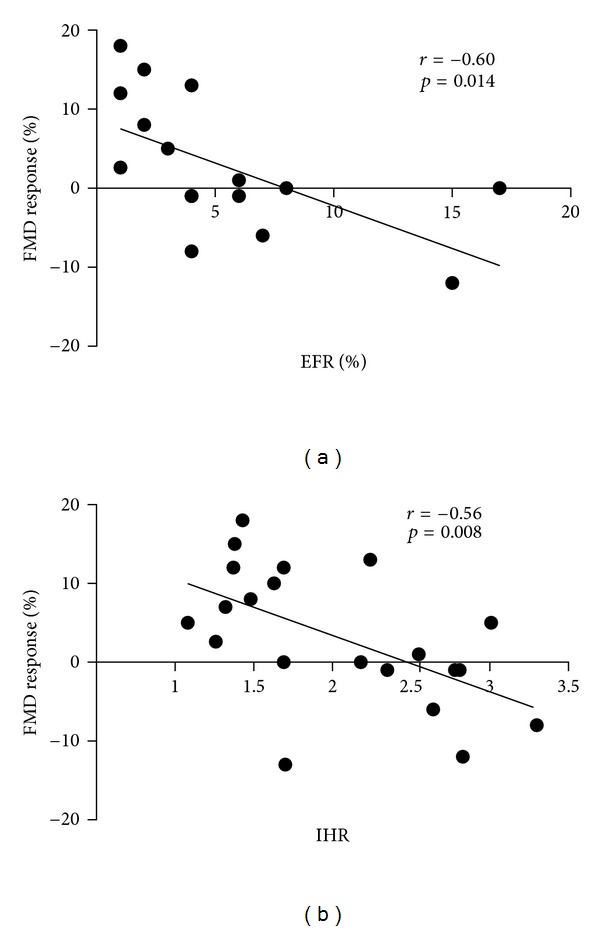
Negative correlation of flow-mediated dilation (FMD) response with Framingham risk score (a) and with baseline reactive hyperemia index (b).

FMD response was inversely correlated with Framingham risk score (*r* = −0.60, *P* = 0.014), baseline FMD (*r* = −0.54, *P* = 0.011), baseline RHI (*r* = −0.56, *P* = 0.008; Figure 1), and aortic pulse pressure (*r* = −0.43, *P* = 0.050). However, after multiple linear regression analysis, only the Framingham risk score and baseline RHI were independently associated with FMD response.

## 4. Discussion

This study demonstrated that treated hypertensive subjects responding to 7-day dark chocolate intakes with improved endothelial function and reduced blood pressure were younger and had less cardiovascular risk than nonresponders.

In contrast to our study, other protocols commonly included only naive hypertensive patients with never-treated raised BP levels or a washout period with discontinuation of drug therapy before the intervention with chocolate [11, 17–19]. Both groups had similar baseline blood pressure and metabolic profiles, but responders had lower baseline FMD and RHI than nonresponders, much as reported by Grassi et al. [18] who described baseline FMD around 8%. Moststudiesshowlower valuesthan these observed in the presentstudy [11, 17, 19], suggesting that those with compromised vascular functionare more responsive tochocolate. Moreover, the higher brachial and aortic pulse pressure among nonresponder patients might indicate increased vascular stiffness in these subjects and could be associated with resistance to favorable effects of cocoa.

 Nonresponders were older and had higher cardiovascular risk scores than responders as reported previously in patients of similar ages to those in our study [21]. Recently, Muniyappa et al. also found a significant improvement of 2.3% in endothelial function in hypertensive patients without drug therapy after consumption of a cocoa drink for fifteen days [19]. The consumption of 100 g of dark chocolate showed benefits in vascular function in healthy subjects after acute consumption of 3 hours, reflected by 1.43% improvement in FMD and 7.8% reduction in augmentation index. These findings also confirmed that chocolate can be beneficial to vascular health [22]. The results of our study showed no improvement in central hemodynamic parameters after consumption of dark chocolate. However, the group that did not respond in FMD, although presenting a good baseline endothelial function, presented significantly greater values of central and peripheral pulse pressure than those in responder group. These findings could indicate increased vascular stiffness among nonresponder patients. The small decrease in FMD after consumption of dark chocolate in nonresponder group was not clinically significant because it remained in the normal range of a good endothelial function. On the other hand, the responder group had a significant increase in FMD which was also clinically relevant. Patients in this group varied from 8.4% (before chocolate) to 16.6% (after chocolate) in FMD, demonstrating a clear improvement in endothelial function.

To date, the gold standard noninvasive technique for measuring endothelial function in clinical studies is the FMD of the brachial artery [23, 24]. It has been recently shown that PAT, which can detect abnormalities in the amplitude of the wave digital pulse, is significantly associated with FMD [25], and we used both methods. However, neither group showed effects on RHI. This discrepancy might reflect the short intervention period. Positive effects are reported on PAT after 5-day cocoa supplementation, probably reflecting increased nitric oxide bioavailability with cocoa [26].

Our responders also showed reductions in blood pressure after eating chocolate as others have with untreated hypertension and in longer studies [11, 17, 20]. Furthermore the reductions being reported, though relatively small, are clinically significant, since it has been found that a reduction of 3 mmHg in systolic BP can reduce relative risk of death from stroke by 8%, from cardiovascular disease in general by 5%, and overall all-cause mortality by 4% [27]. In our study, the responder group also demonstrated a reduction in aortic systolic blood pressure and pulse pressure, although not reaching statistical significance. This result may suggest a less prominent effect of dark chocolate on vascular stiffness parameters.

 In a recent prospective study, Buijsse et al. investigated the effects of chocolate consumption on BP and incidence of cardiovascular disease after 8-year followup and found chocolate eating reduced cardiovascular risk, largely due to the reductions in blood pressure [28]. A new meta-analysis supports the findings for benefit with chocolate consumption [14], which may reflect increased polyphenol intakes which likely improves vascular health through increased nitric oxide bioavailability, inhibition of angiotensin-converting enzyme stimulating the production of vasodilator factors, and improvement of insulin sensitivity which are the most studied pathways to explain the effect of epicatechin, a monomer of polyphenols, on vascular function [13, 29–32]. Another recent meta-analysis showed chocolate usage was associated with reductions in risk of cardiovascular disease by 31%, of diabetes by 37%, and of stroke by 29% [33], and protection is greater with dark than other chocolates [34]. 

Our study had some limitations, especially concerning the sample size and lack of a control group. Nevertheless, the findings are in line with other recent observations. There is as yet no way to identify those who will benefit from dark chocolate consumption, but the present study provides data that could be examined in larger randomized controlled studies for the selection of subjects likely to benefit from increased intakes of dark chocolate.

## 5. Conclusion

In conclusion, the intake of dark chocolate significantly improved endothelial function and reduced blood pressure in some individuals who were younger hypertensive patients with impaired endothelial function in spite of lower cardiovascular risk.

## Figures and Tables

**Table 1:** Baseline characteristics of study population and responder (RESP) and nonresponder (N-RESP) groups.

Parameters	Total sample (*n* = 21)	RESP (*n* = 12)	N-RESP (*n* = 9)	*P* value
Age, years	57 ± 8	54 ± 8	61 ± 6*	0.037
Framingham risk score, %	5.3 ± 4.7	2.5 ± 1.8	8.1 ± 5.1*	0.011
Body mass index, Kg/m²	28.2 ± 4.5	29.7 ± 5.2	26.1 ± 2.9	0.065
Systolic BP, mmHg	141 ± 11	140 ± 12	142 ± 8	0.718
Diastolic BP, mmHg	82 ± 9	85 ± 7	79 ± 9	0.147
Pulse pressure, mmHg	58 ± 9	55 ± 9	63 ± 5	0.041
Mean arterial pressure, mmHg	101 ± 9	103 ± 8	100 ± 9	0.417
Total cholesterol, mmol/L	5.56 ± 1.21	5.30 ± 0.77	5.84 ± 1.52	0.355
HDL-cholesterol, mmol/L	1.24 ± 0.36	1.27 ± 0.31	1.21 ± 0.41	0.771
LDL-cholesterol, mmol/L	3.46 ± 0.72	3.31 ± 0.67	3.62 ± 0.77	0.411
Triglycerides, mmol/L	1.56 ± 1.13	1.41 ± 0.53	1.70 ± 1.55	0.594
Fasting glucose, mmol/L	4.72 ± 0.5	4.72 ± 0.61	4.67 ± 0.39	0.864
Flow-mediated dilation, %	10.7 ± 5.5	8.4 ± 5.9	13.8 ± 2.8*	0.023
Reactive hyperemia index, units	2.03 ± 0.71	1.70 ± 0.63	2.47 ± 0.51**	0.006
Augmentation pressure, mmHg	15 ± 7	15 ± 7	16 ± 5	0.869
Augmentation index, %	32 ± 11	34 ± 12	29 ± 9	0.343
Aortic systolic BP, mmHg	130 ± 15	128 ± 18	133 ± 11	0.550
Aortic pulse pressure, mmHg	48 ± 10	44 ± 10	54 ± 6*	0.021

Results are expressed as mean ± SD. BP: blood pressure; HDL: high density lipoprotein; LDL: low-density lipoprotein. *P < 0.05 and **P < 0.01 versus responder group.

**Table 2:** Clinical and vascular parameters in responder (RESP) and nonresponder (N-RESP) groups before and after chocolate consumption.

Parameters	RESP group (*n* = 12)			N- RESP group (*n* = 9)		
	Before chocolate	After chocolate	*P *	Before chocolate	After chocolate	*P *
Weight (Kg)	78.8 ± 11.9	78.3 ± 11.4	0.185	71.2 ± 16.1	71.5 ± 16.3	0.403
SBP (mmHg)	140 ± 13	131 ± 10	0.015	142 ± 8	142 ± 10	1.000
DBP (mmHg)	85 ± 7	82 ± 8	0.050	79 ± 9	83 ± 10	0.276
PP (mmHg)	55 ± 9	48 ± 5	0.084	63 ± 5	60 ± 7	0.313
MAP (mmHg)	103 ± 9	98 ± 9	0.007	100 ± 9	102 ± 10	0.368
FMD (%)	8.4 ± 5.9	16.6 ± 8.2	<0.001	13.8 ± 2.8	11.3 ± 4.2	0.030
RHI (units)	1.70 ± 0.63	1.87 ± 0.54	0.134	2.47 ± 0.51	2.17 ± 0.42	0.124
AP (mmHg)	15 ± 7	11 ± 5	0.092	16 ± 5	13 ± 10	0.323
Aix (%)	34 ± 13	28 ± 12	0.209	29 ± 9	26 ± 12	0.299
Aortic SBP (mmHg)	128 ± 19	121 ± 9	0.249	133 ± 11	131 ± 18	0.732
Aortic PP (mmHg)	44 ± 10	36 ± 7	0.079	53 ± 6	46 ± 13	0.127

Results are expressed as mean ± SD. SBP: systolic blood pressure; DBP: diastolic blood pressure; PP: pulse pressure; MAP: mean arterial pressure; FMD: flow-mediated dilation; RHI: reactive hyperemia index; AP: augmentation pressure; Aix: augmentation index.
